# Composite microneedle-mediated co-delivery of magnesium hydride–magnolol for androgenetic alopecia

**DOI:** 10.1039/d6ra06529e

**Published:** 2026-10-13

**Authors:** Wenting Wang, Xiaoxia Wang, Xiaojiang Hou, Tao Liu, Da Zhai, Tianyu Cao, Ling Liu

**Affiliations:** a Department of Dermatology, Tangdu Hospital, Fourth Military Medical University Xi'an 710038 China tianyucao1991@outlook.com liuling_derm@163.com; b School of Material Science and Engineering, Shaanxi Key Laboratory of Green Preparation and Functionalization for Inorganic Materials, Shaanxi University of Science and Technology Xi'an 710021 China houxiaojiang@sust.edu.cn

## Abstract

Androgenetic alopecia (AGA) impairs quality of life, yet current pharmacotherapies are hampered by limited efficacy and adverse effects. Microneedle-assisted transdermal co-delivery of bioactive natural products and gaseous agents offers a promising alternative. Herein, we report composite polymer microneedles (MNs) for controlled co-delivery of magnesium hydride (MgH_2_, which generates cytoprotective H_2_ and Mg^2+^*in vivo*) and magnolol (Mag, a polyphenol with anti-inflammatory and antioxidant activities). Drug-loaded particles were fabricated *via* mechanical ball milling and ethanol solvent methods, characterized by XRD, FTIR and SEM, and then incorporated into dissolving PVA:PVP MNs by micromolding with two-step centrifugation. Ball milling yielded superior phase purity and a denser microstructure compared with ethanol, evidenced by distinct XRD peaks and intact FTIR signatures for both Mag and MgH_2_. Optimized MNs exhibited markedly enhanced mechanical strength after drug loading (14.524 N *vs.* 10.729 N). *In vitro* release best followed first-order kinetics (*R*^2^ = 0.99879), reaching 96% at 40 °C or under UV irradiation *versus* 80.7% at ambient conditions. This MgH_2_–Mag MN system combines robust mechanical resilience with thermoresponsive and photoresponsive release, providing a tunable transdermal platform for AGA intervention.

## Abstract 2

Composite MgH_2_-magnolol dissolving microneedles, reinforced by PVA-PVP and loaded with ball-milled drug formulation, enable diffusion-controlled, thermo/UV-responsive release and H_2_/Mg^2+^-magnolol synergy for androgenetic alopecia.

## Graphical abstract

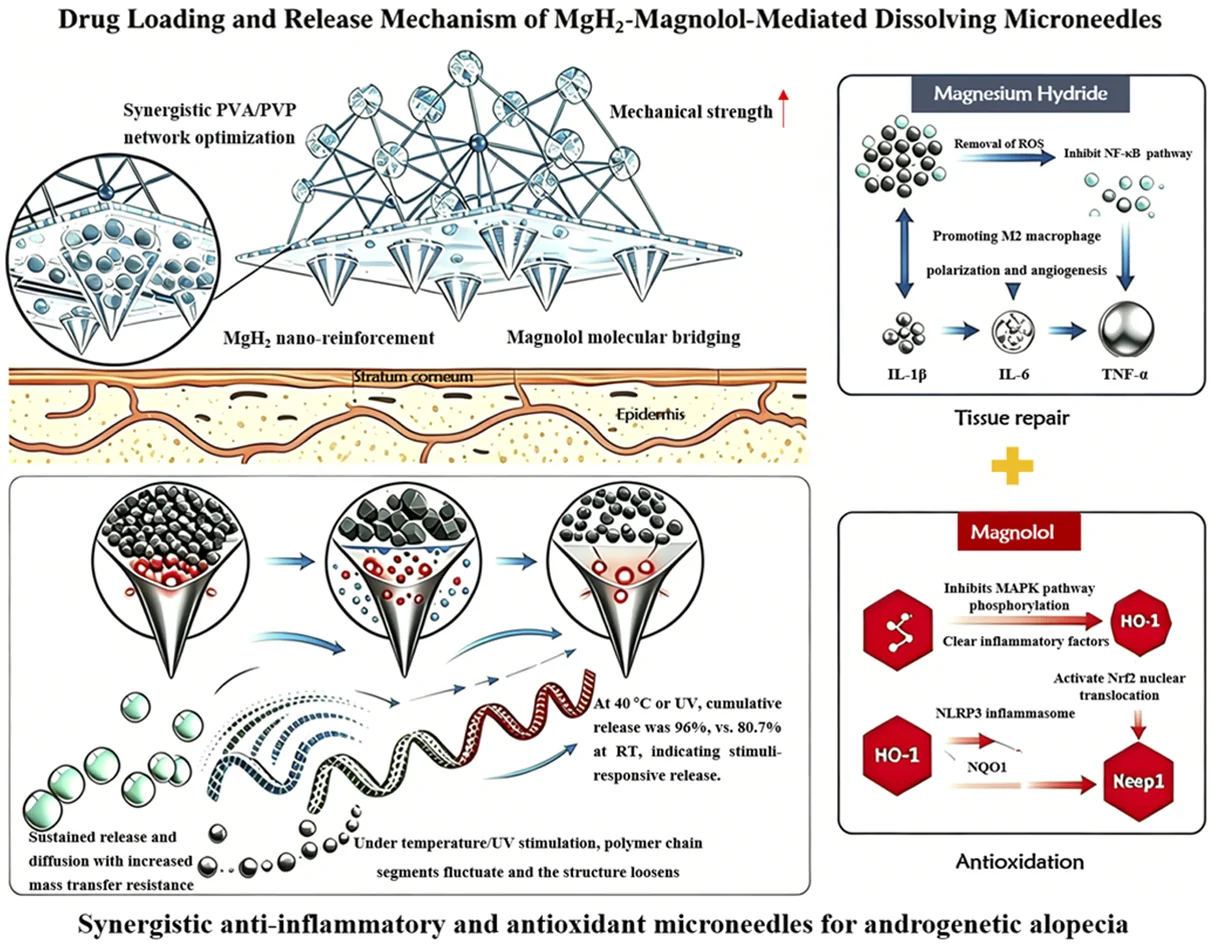

## 1. Introduction

Androgenetic alopecia (AGA) is the most prevalent form of hair loss worldwide, affecting up to 80% of men and 50% of women over their lifetime.^1^ In recent years, an increasing incidence of AGA has been observed among younger populations, including pediatric and adolescent cohorts.^2^ This underscores the urgent need to elucidate its pathogenesis and develop effective therapeutic interventions.

Although AGA has traditionally been characterized as a non-inflammatory, androgen-driven disorder, emerging evidence indicates that its pathogenesis primarily reflects homeostatic imbalance of the perifollicular microenvironment driven by elevated oxidative stress and impaired vascularization.^3,4^ Recent comprehensive reviews^5,6^ establish that oxidative stress and chronic inflammation, together with the DHT/PGD_2_ loop and scalp dysbiosis, independently suppress Wnt/β-catenin signaling and promote follicular miniaturization, as evidenced by elevated ROS, lipid peroxide-induced catagen entry, and perifollicular fibrosis in AGA, while ROS mediates androgen-induced TGF-β1 secretion that is abrogated by *N*-acetylcysteine.^7^ Mechanistically, H_2_ scavenges hydroxyl radicals and peroxynitrite to resolve oxidative stress and promote M2 macrophage polarization, Mg^2+^ enters dermal papilla cells *via* TRPM7 to activate PI3K/Akt and VEGF-mediated angiogenesis,^8^ and magnolol and its isomer honokiol activate Wnt/β-catenin and downregulate TGF-β1, thereby counteracting DHT-driven fibrosis.^9^ Thus, H_2_, Mg^2+^, and magnolol target the oxidative and inflammatory network through complementary and interconnected mechanisms, and these mechanistic and evidential details have been incorporated into the revised Introduction with appropriate citations. During pathological progression, excessive accumulation of dihydrotestosterone (DHT) induces the generation of reactive oxygen species (ROS), which serves as a key initiating event that not only directly damages dermal papilla cells but also impedes the normal transition of the hair follicle cycle from telogen to anagen by suppressing the Wnt/β-catenin signaling pathway.^10,11^ Concurrently, local ischemia and downregulated expression of angiogenic factors such as vascular endothelial growth factor (VEGF) lead to severe insufficiency in nutrient supply to hair follicles, further exacerbating follicular miniaturization.^12–16^ Clinical studies have further corroborated these findings, demonstrating that patients with AGA exhibit elevated serum total oxidant status (TOS), coupled with reduced total antioxidant status (TAS) and diminished endogenous thiol levels, a profile indicative of a systemic shift toward a pro-oxidative milieu.

Notably, although AGA has long been regarded as a non-inflammatory, non-scarring alopecia, recent studies have revealed the presence of occult inflammatory responses within its microenvironment, characterized histopathologically by perifollicular sheath thickening, mast cell degranulation and T-cell infiltration^17–19^ are spatially localized to the follicular bulge region, where they directly compromise the functional integrity of hair follicle stem cells (HFSCs). This low-grade chronic inflammation not only promotes perifollicular fibrosis but also forms a vicious cycle with oxidative stress and impaired vascularization, collectively compromising nutrient delivery to the follicle. The intricate interplay among oxidative stress, impaired angiogenesis, and occult inflammation disrupts the hair growth cycle, drives follicular miniaturization, and promotes premature entry into telogen.

Given these complex pathological features, monotherapy often proves insufficient, necessitating multi-target synergistic intervention as a key therapeutic strategy. The present study proposes a novel combination regimen of magnolol and magnesium hydride. Magnolol, a natural polyphenolic compound derived from *Magnolia officinalis*, significant anti-inflammatory, antioxidant, and antibacterial properties.^9,20^ Magnolol and its structural isomer honokiol constitute the principal bioactive biphenyl constituents of *Magnolia officinalis*, and they display complementary pharmacological activities in anti-inflammatory and antioxidant modulation. Previous studies have demonstrated that honokiol and its liposomal formulations can accelerate the transition of hair follicles from telogen to anagen by activating the Wnt/β-catenin signaling pathway and downregulating TGF-β1 expression, thereby reversing DHT-mediated follicular miniaturization at the molecular level. These findings further substantiate the therapeutic potential of magnolol-class polyphenols in hair follicle cycle regulation and provide a structure–activity rationale for the selection of magnolol as a combinatorial intervention component in the present study. Magnesium hydride serves as a dual donor of magnesium ions and hydrogen gas (H_2_): Mg^2+^ enters cells *via* the TRPM7 channel and activates the PI3K/Akt signaling pathway, which in turn upregulates the expression of proangiogenic factors such as VEGF and promotes angiogenesis, while the released hydrogen gas enables selective scavenging of highly cytotoxic hydroxyl radicals (˙OH) and peroxynitrite anions (ONOO^−^), thereby achieving precise antioxidant activity.^21^ The rationale for this combination lies in its potential to reverse the imbalanced follicular microenvironment through synergistic anti-inflammatory, antioxidant, and immunomodulatory actions.

To achieve efficient transdermal delivery of the therapeutic agents, this study employs dissolvable microneedle (DMN) technology.^22–25^ Although DMNs offer inherent advantages including facile fabrication, high drug loading capacity, and convenient self-administration, conventional single-polymer microneedles frequently suffer from limitations such as insufficient mechanical strength to ensure reliable penetration of the stratum corneum and suboptimal drug release profiles.^26–29^ Current AGA therapies including minoxidil, finasteride, and microneedling are limited by poor percutaneous absorption, relapse, systemic endocrine effects, and single target or purely physical action, leaving oxidative stress, perifollicular inflammation, and established follicular miniaturization unaddressed.^30^ To overcome these limitations, this study systematically screens polymeric materials with superior mechanical properties and optimizes the polymer to drug ratio to fabricate microneedle arrays that combine high drug loading with adequate mechanical robustness, based on the hypothesis that integrating magnolol and MgH_2_ into a dissolvable microneedle delivery system creates a multitarget synergistic composite platform combining a bioactive compound derived from traditional Chinese medicine with Mg ions and hydrogen gas, thereby enabling precision treatment of perifollicular inflammation, effective modulation of the follicular microenvironment, and multiresponsive sustained drug release as a novel paradigm for combination therapy in AGA. This MgH_2_ and magnolol dissolvable microneedle system integrates physical follicular activation, *in situ* H_2_ antioxidant protection, and magnolol mediated antiinflammatory and antioxidant effects *via* Wnt/β-catenin activation and TGF-β1 downregulation,^31^ enabling release responsive to both temperature and light for multitarget synergy, localized delivery, precision dosing, and improved compliance through a painless patch regimen.

## 2. Materials and methods

### 2.1 Materials

MgH_2_ purity ≥98% was obtained by Dalian Funde Jinyu Clean Energy Co., Ltd (China). Magnolol purity ≥98% was obtained by Shanghai Dibai Biotechnology Co., Ltd (China), PVP(K90) and PVA (98–99% hydrolyzed, MW 31–50 kDa) were obtained by Shanghai Macklin Biochemical Technology Co., Ltd (China), PBS buffer (pH = 7.4) was obtained Zancheng Tianjin Technology Co., Ltd (China).

### 2.2 Preparation

#### 2.2.1 Preparation of the MgH_2_–magnolol drug formulation

Composite particles were prepared by ethanol solvent deposition and mechanical ball milling. In the deposition method, magnolol dissolved in anhydrous ethanol was added dropwise to magnesium hydride powder under an inert atmosphere with stirring at 500 rpm for 24 h to enable physical adsorption. The product was washed with ethanol and dried at 60 °C for 24 h. In the ball milling method, magnolol and magnesium hydride at a 1 to 1 mass ratio were milled in a planetary ball mill at 600 rpm for 2 h with a ball to powder mass ratio of 20 to 1 under argon protection. The final composite particles were collected in a glove box to prevent oxidation.

#### 2.2.2 Fabrication of drug-free dissolving polymer microneedles

Systematic screening compared pure polyvinylpyrrolidone (PVP), pure polyvinyl alcohol (PVA), and PVA:PVP (1 : 1) microneedle arrays. The 1 : 1 blend was preliminarily selected without gradient optimization because only its fracture force (10.729 N) fell within the literature-validated range (9.56 to 56.38 N), and MgH_2_–magnolol-loaded arrays exhibited the highest mechanical strength at 250 mg. Arrays were fabricated by micromolding with a polydimethylsiloxane (PDMS) mold and two-step centrifugation: tip solution filling at 8000 rpm for 10 min, pre-drying at 60 °C for 60 min, backing layer addition and centrifugation at 2000 rpm for 5 min, and final vacuum drying for 24 h before peeling to obtain intact arrays.

#### 2.2.3 Fabrication of MgH_2_–magnolol-mediated dissolving microneedles

Microneedle arrays were fabricated using a PDMS mold *via* a two-step centrifugation micromolding method. The backing layer solutions were prepared by PVA:PVP (1 : 1) in deionized water. Magnesium hydride magnolol composite particles were added to the backing solution and ultrasonicated at 200 W for 15 min to obtain the tip formulation. The tip formulation was dispensed onto the mold and centrifuged at 4000 rpm for 10 min to fill the cavities. The backing layer solution was then applied and centrifuged at 2000 rpm for 5 min. After vacuum drying for 24 h, the mold was peeled to obtain drug loaded microneedle arrays.

### 2.3 Characterization

#### 2.3.1 Phase compositions

Appropriate amounts of magnesium hydride magnolol particle powders prepared by the ethanol solvent method and the mechanical ball milling method were taken for X-ray diffraction analysis. The scanning speed was 8° min^−1^ and the scanning range was from 10° to 80°.

#### 2.3.2 Morphology

Scanning electron microscopy was employed to examine the surface morphology and structural integrity of the fabricated microneedle arrays. Samples were sputter coated with gold, mounted at a 45° tilt angle, and observed under a Zeiss Gemini 300 field emission scanning electron microscope.

#### 2.3.3 Chemical compositions

FTIR spectroscopy was used to characterize sample chemical composition and assess drug polymer interactions. KBr powder was dried at 105 °C for 5 hours, ground in an agate mortar, and pressed into a blank pellet for background reference. Each of the four samples was mixed with KBr and compressed into pellets following the same procedure. FTIR spectra were recorded on a Bruker VERTEX 70 spectrometer.

#### 2.3.4 Mechanical property

Compression testing was conducted using a texture analyzer (TBUTA 23, Shanghai Tengba Instrument Co., Ltd, China) to assess the mechanical strength of microneedle arrays. Samples were fixed with tips upward on the stage using double-sided tape. After calibration, a vertical compressive load was applied at 0.05 mm s^−1^ with a maximum displacement of 400 µm. Force–displacement data were recorded, and a force–displacement curve was plotted to evaluate the mechanical properties. To visually verify the skin penetration capability of the microneedle arrays, trypan blue staining was employed to evaluate the insertion efficiency of PVA:PVP (1 : 1) microneedles on *ex vivo* porcine skin. Fresh *ex vivo* porcine skin, obtained by the authors from Yonghui Supermarket Huayangcheng Store in Baqiao District, Xi'an, was used for *in vitro* piercing experiments of the microneedle system. The microneedle arrays were manually pressed onto the skin for 30 s and subsequently removed. The skin surface was then stained with trypan blue solution for 5 min and thoroughly rinsed with PBS.

#### 2.3.5 Drug loading and releasing

Drug loading content of magnolol and magnesium ions in each microneedle patch was determined by UV-Vis spectrophotometry using a Cary 5000 instrument. For *in vitro* release, each patch was placed in 10 mL PBS under magnetic stirring at ambient temperature. At 10, 20, 30, 40, 50, 60, 70, 80, and 90 min, 1 mL of release medium was withdrawn and replaced with an equal volume of fresh PBS. The UV absorbance of each sample was measured to calculate the released drug concentration and cumulative release percentage.

## 3. Results and discussions

### 3.1 Phase compositions of MgH_2_–magnolol drug formulation

XRD analysis was performed to investigate the phase constitution of magnesium hydride and magnesium hydride magnolol composite particles prepared *via* different routes (Fig. 1). The diffraction pattern of pristine magnesium hydride (Fig. 1a) exhibits sharp peaks consistent with tetragonal α-MgH_2_ (JCPDS No. 74-0934), with major reflections at 2*θ* approximately 27°, 37°, 44°, 52°, 60°, and in the 67–75° region. The flat baseline and the absence of detectable impurity signals such as MgO or metallic Mg confirm the high crystallinity and phase purity of the starting material. Magnolol (Fig. 1b) exhibits a distinct diffraction pattern attributable to its monoclinic *P*2_1_/*c* crystal structure, characterized by dense low-angle reflections in the 2*θ* range of approximately 12–25° that attenuate rapidly beyond 30°. For the ethanol solvent-deposited composite (Fig. 1c), only MgH_2_ diffraction signals appear, with no detectable magnolol peaks, indicating that magnolol is amorphous, highly dispersed, or below the XRD detection limit, consistent with solvent-induced amorphization. In contrast, the ball-milled sample (Fig. 1d) shows characteristic peaks of both MgH_2_ and magnolol, confirming biphasic coexistence and preserved magnolol crystallinity, in line with reported ball-milled MgH_2_ composites in which the hydride framework remains crystalline while organic additives are incorporated without loss of phase identity. These results indicate that ethanol solvent deposition reduces magnolol crystallinity, whereas ball milling preserves both phases and enables precise regulation of phase composition and interfacial architecture. Comparison of Fig. 1c with Fig. 1a and b further confirms that ball milling effectively composites α-MgH_2_ and magnolol while retaining their phase features; therefore, all subsequent experiments used the ball-milled composite.

**Fig. 1:**
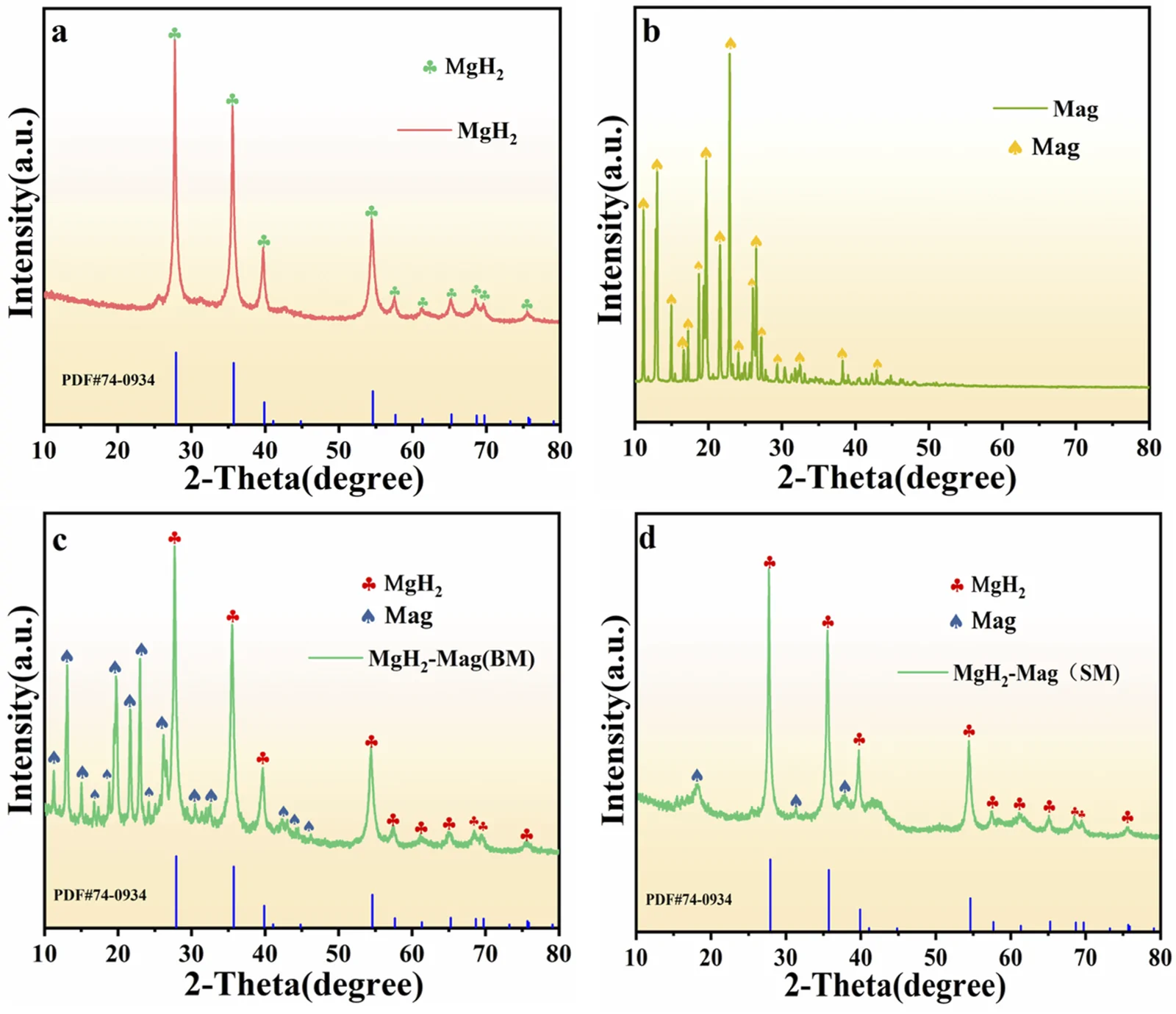
XRD diffraction patterns. (a) MgH_2_, (b) magnolol (Mag), (c) MgH_2_–magnolol (BM), (d) MgH_2_–magnolol (SM).

### 3.2 Chemical analysis of MgH_2_–magnolol drug formulation

FTIR characterization was performed on pure MgH_2_, pure magnolol, and the composites obtained by ethanol solvent deposition and mechanical ball milling to elucidate their chemical composition and functional group states (Fig. 2). Pure MgH_2_ exhibited characteristic H^−^ absorptions, with Mg–H stretching bands in the 1150–1500 cm^−1^ region and Mg–H bending bands in the 759–1000 cm^−1^ region, while pure magnolol displayed its phenolic O–H stretching vibration and aromatic C=C skeletal stretching vibration. In the ethanol solvent-deposited composite (Fig. 2a), the H^−^ absorption of MgH_2_ was markedly altered, the O–H peak of magnolol diminished significantly and blue-shifted to approximately 3696 cm^−1^, and the remaining magnolol characteristic peaks nearly disappeared, suggesting that the phenolic hydroxyl groups of magnolol engage in physical interactions such as hydrogen bonding with ethanol molecules or the MgH_2_ surface, or that magnolol is present at an exceedingly low loading with high dispersion. In contrast, the ball-milled composite (Fig. 2b) concurrently retained the H^−^ absorption of MgH_2_ and multiple magnolol signatures including C–O stretching and C–H deformation vibrations, with peak profiles of both components remaining largely intact, indicating that ball milling does not significantly damage the functional group structures of either component. Both composites exhibited an absorption near 1262 cm^−1^ similar to pure MgH_2_, indicating that the preparation route does not fundamentally alter the bulk MgH_2_ structure, although the severe attenuation of magnolol signals and the pronounced O–H shift in the ethanol-processed sample imply a substantially different state and content of magnolol compared with the ball-milled sample. FTIR results are consistent with the XRD data, showing that ball milling preserves the crystallinity and functional groups of magnolol, whereas ethanol processing leads to its dispersion or functional group perturbation. This indicates that the preparation route governs the chemical states of the MgH_2_–Mag composite: ball milling enables physical mixing without forming new bonds or causing drug degradation, thereby establishing it as a chemically mild and safe strategy.

**Fig. 2:**
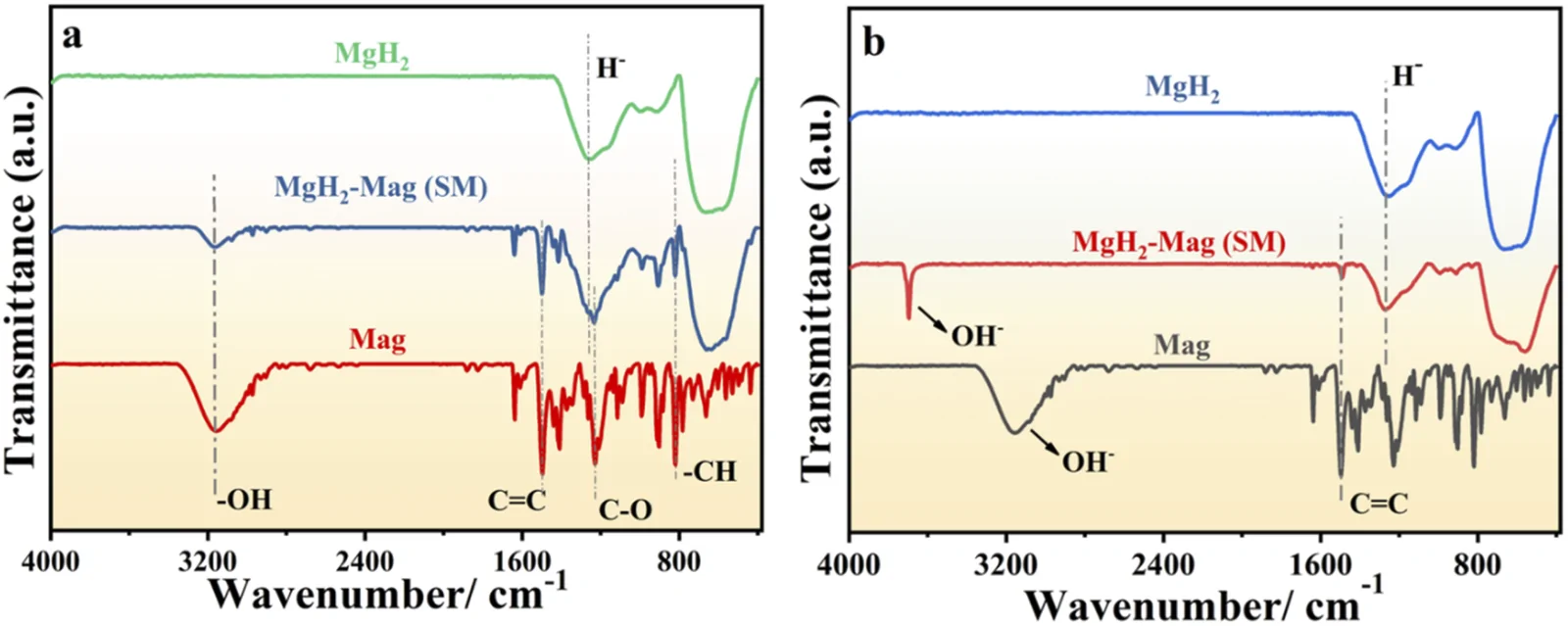
FTIR spectra of MgH_2_–magnolol from different preparation methods: (a) ethanol solvent method (SM), (b) ball milling (BM).

### 3.3 Morphology of MgH_2_–magnolol drug formulation

Scanning electron microscopy was employed to compare the micromorphology of pristine magnesium hydride, pristine magnolol, and their composites prepared *via* two methods (Fig. 3). Pristine magnesium hydride appears as irregular agglomerates with rough surfaces and abundant micropores (Fig. 3a–c), which are attributed to mechanical fracturing during ball milling and are expected to provide adsorption sites for enhanced drug loading. Pristine magnolol exhibits a regular plate-like morphology with smooth edges (Fig. 3d–f), reflecting anisotropic crystal growth, and may serve as a planar support or encapsulating matrix for magnesium hydride within the composite. The composite prepared by mechanical ball milling shows a compact, uniform morphology with intimately interwoven magnesium hydride and magnolol phases (Fig. 3g–i), which is anticipated to prolong drug diffusion pathways and sustain release.^32,33^

**Fig. 3:**
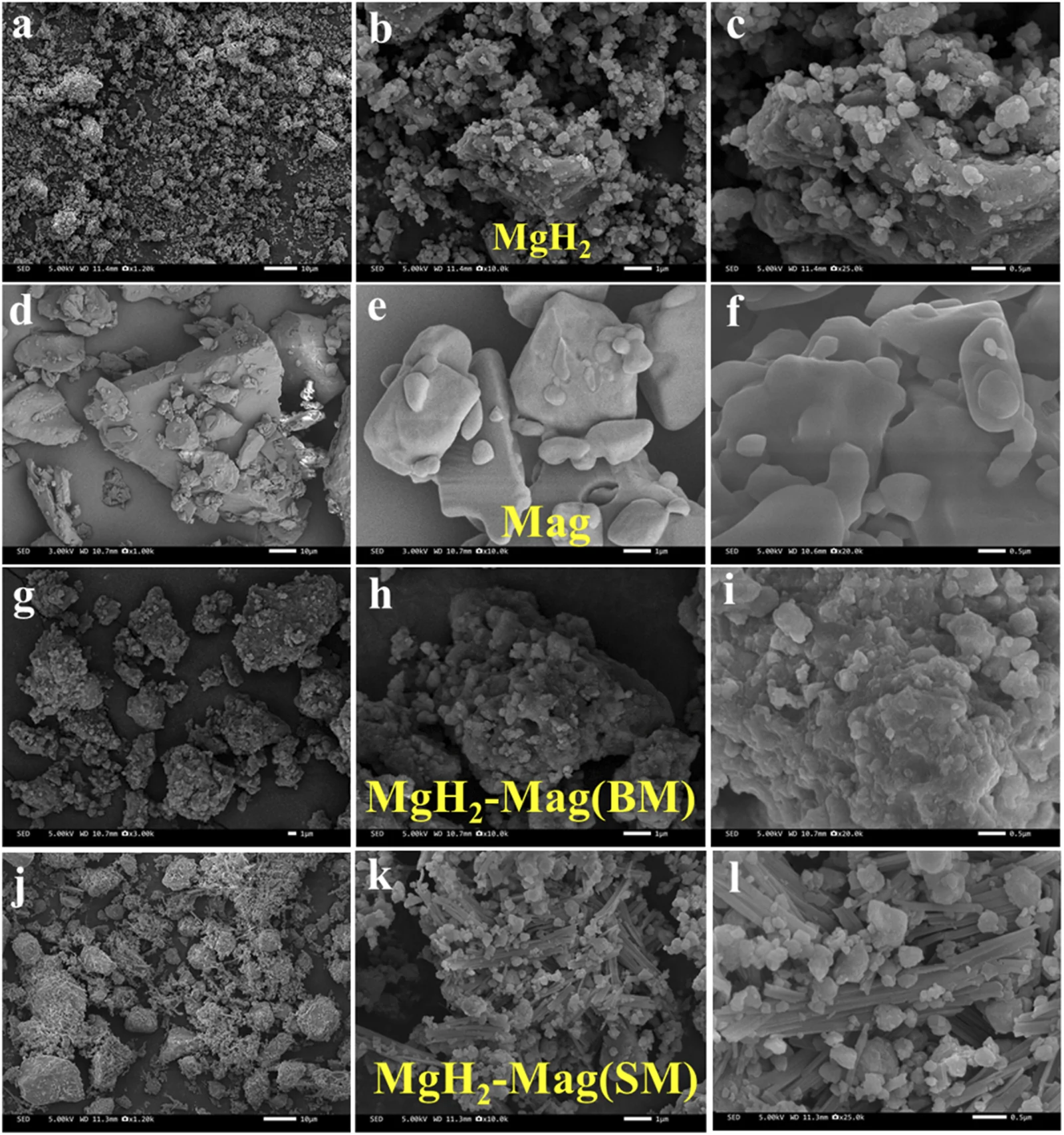
Morphology of MgH_2_, magnolol and MgH_2_–magnolol release unit prepared by ball milling (BM) and ethanol solvent method (SM) methods. (a–c) MgH_2_, (d–f) magnolol, (g–i) MgH_2_–magnolol (BM), (j–l) MgH_2_–magnolol (SM).

In contrast, the ethanol solvent deposition method yields a looser mixture of irregular magnesium hydride particles and plate-like magnolol with distinct interfacial contact but limited integration (Fig. 3j–l). The compact, interwoven ball-milled MgH_2_–Mag composite acts as a physical barrier-type sustained-release matrix through three coupled mechanisms: ball milling embeds magnolol microcrystals within a dense MgH_2_ skeleton, reducing porosity and increasing tortuosity, which lowers the effective diffusion coefficient and retards release;^34,35^ the hydrophobic MgH_2_ surface and low porosity impede capillary wetting, causing a wetting lag and shifting release to layer-by-layer erosion that eliminates burst release; and milling-induced lattice defects and interfacial distortions create mechanical interlocking that restricts drug dissociation and enhances matrix retention.^36^ This structure-function coupling contrasts with the rapid-release design of solvent-deposited composites based on amorphization and hydrogen bonding, making the two strategies suitable for sustained *versus* rapid-onset applications. Ball milling preserves the MgH_2_ framework and crystallinity, retains magnolol structural integrity, and yields a more homogeneous and compact morphology, as supported by FTIR analysis. Accordingly, subsequent microneedle drug-loading experiments will uniformly adopt ball-milled MgH_2_–Mag composite particles as the drug-loading matrix.

### 3.4 Morphology and mechanical properties of MgH_2_–magnolol-mediated dissolving microneedles

SEM was employed to characterize the morphology of the dissolving polymeric microneedles (Fig. 4). The drug-free microneedle array exhibited an orderly arrangement with a square base and pyramidal needle bodies, and each needle had a height of 550 µm, a base width of 200 µm, an inter-base spacing of 200 µm, and a tip-to-tip distance of 400 µm, demonstrating excellent array uniformity and a well-controlled fabrication process. At higher magnification, the microneedle surfaces displayed distinct lamellar textures with sharp edges and no apparent defects, while the needle tips exhibited favorable morphology with only slight bending, presumably resulting from friction with the aluminum foil pouch during storage, and this uniform layered architecture conferred satisfactory initial morphological stability to the microneedles (Fig. 4a–c). SEM at increasing magnifications revealed the drug distribution and mechanical behavior of drug-loaded microneedles. At 30 000×, the needles were orderly with uniform spacing and an intact structure; at 100 000×, small protrusions from residual drug after needle-tip solution removal appeared on the base; at 250 000×, drug-derived protrusions on the needle-tip surfaces confirmed successful loading (Fig. 4d–f). These protrusions increased the drug contact area, facilitating release and permeation and enhancing utilization efficiency.^37^ Mechanically tested arrays showed needle-tip bending without fracture, indicating excellent toughness and reduced breakage risk during skin penetration (Fig. 4g–i).

**Fig. 4:**
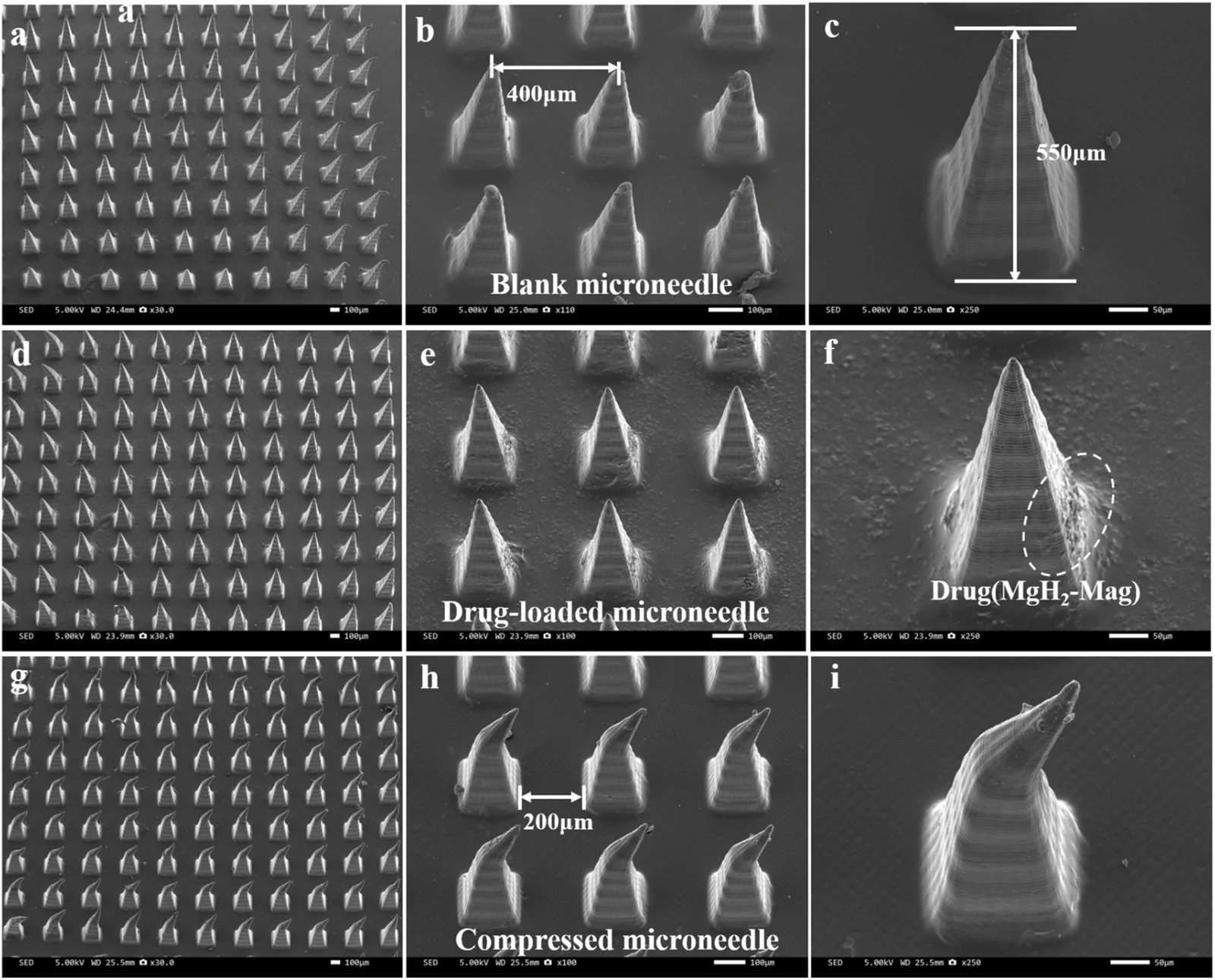
Micromorphology of microneedles (a–c) blank microneedle without release unit, (d–f) drug-loaded microneedle with release unit, (g–i) compressed microneedle.

To evaluate the mechanical properties of the microneedles and identify the formulation with optimal mechanical strength, microneedles of different compositions were subjected to mechanical testing using a texture analyzer (Fig. 5). The tested samples included polyvinyl alcohol microneedles, polyvinylpyrrolidone microneedles, polyvinylpyrrolidone polyvinyl alcohol composite microneedles, and microneedles with varying drug loadings. The force and displacement data during axial compression were recorded, and force–displacement curves were constructed. Within the displacement range of 0 to 400 µm, the force–displacement curves for all microneedle groups exhibited a continuous and smooth trend, although the forces sustained by each group showed marked differences. Among the formulations, the polyvinylpyrrolidone polyvinyl alcohol composite microneedles sustained the highest force at equivalent displacements. At the maximum displacement of 400 µm, this group sustained a force of 10.729 N (Fig. 5a). Previous studies have demonstrated that the overall compressive force of PVA/PVP composite microneedle arrays typically ranges from 9.56 to 56.38 N, within which the arrays have been confirmed to effectively penetrate the skin barrier and achieve transdermal delivery.^38^ Based on this empirical threshold, only the PVA:PVP (1 : 1) formulation yielded an overall fracture force (10.729 N) within this range. The force–displacement curves also varied with drug loading. Microneedles with a drug loading of 250 mg sustained the highest force during testing, reaching 14.524 N, followed by those with a 500 mg drug loading. Microneedles with a 150 mg drug loading fractured during compression and exhibited the lowest sustained force (Fig. 5b). The improved mechanical strength of the drug-loaded PVA:PVP microneedles relative to their unloaded counterparts arises from multiple synergistic physicochemical interactions between the drug components and the polymer matrix. First, the drug molecules infiltrate and occupy the intrinsic microvoids and structural defects within the microneedle body, effectively densifying the material and reducing local stress concentrators. Second, these molecules engage in hydrogen bonding and other non-covalent associations with the polymer chains, creating a reinforcing phase that enhances the overall rigidity of the composite. In this specific system, three cooperative mechanisms are primarily responsible for the observed reinforcement. The hydrogen bonds formed between PVA and PVP generate a dense, well-interconnected network that provides a robust structural backbone. The uniformly dispersed MgH_2_ nanoparticles act as rigid inorganic fillers, efficiently bearing compressive loads and increasing the stiffness of the composite. Meanwhile, magnolol, the active pharmaceutical ingredient, contributes additional crosslinking sites through its phenolic hydroxyl groups, which further tighten the polymer network *via* supplementary hydrogen bonds. Collectively, these filling, crosslinking, and filler-reinforcement effects significantly elevate the mechanical performance of the microneedles, ensuring sufficient hardness and integrity for reliable skin penetration and subsequent drug release.^39,40^ The resulting staining pattern revealed a clear and uniform array of blue spots whose spatial distribution was in precise registry with the 15 × 15 microneedle geometry. The spots exhibited regular morphology and sharp boundaries, with no discernible diffusion or irregular staining, indicating that the majority of needles successfully penetrated the stratum corneum and created open microchannels (Fig. 5c and d). Notably, trypan blue (molecular weight ≈ 961 Da) is incapable of passively traversing the intact stratum corneum; thus, staining is contingent upon the presence of microchannels pre-formed by microneedle insertion.^41,42^ Consistent with this, application of trypan blue to intact, unpenetrated skin under identical conditions yielded no residual staining. The observed staining pattern therefore provides direct evidence that the microneedle arrays developed in this study are capable of efficient and consistent penetration of the stratum corneum.

**Fig. 5:**
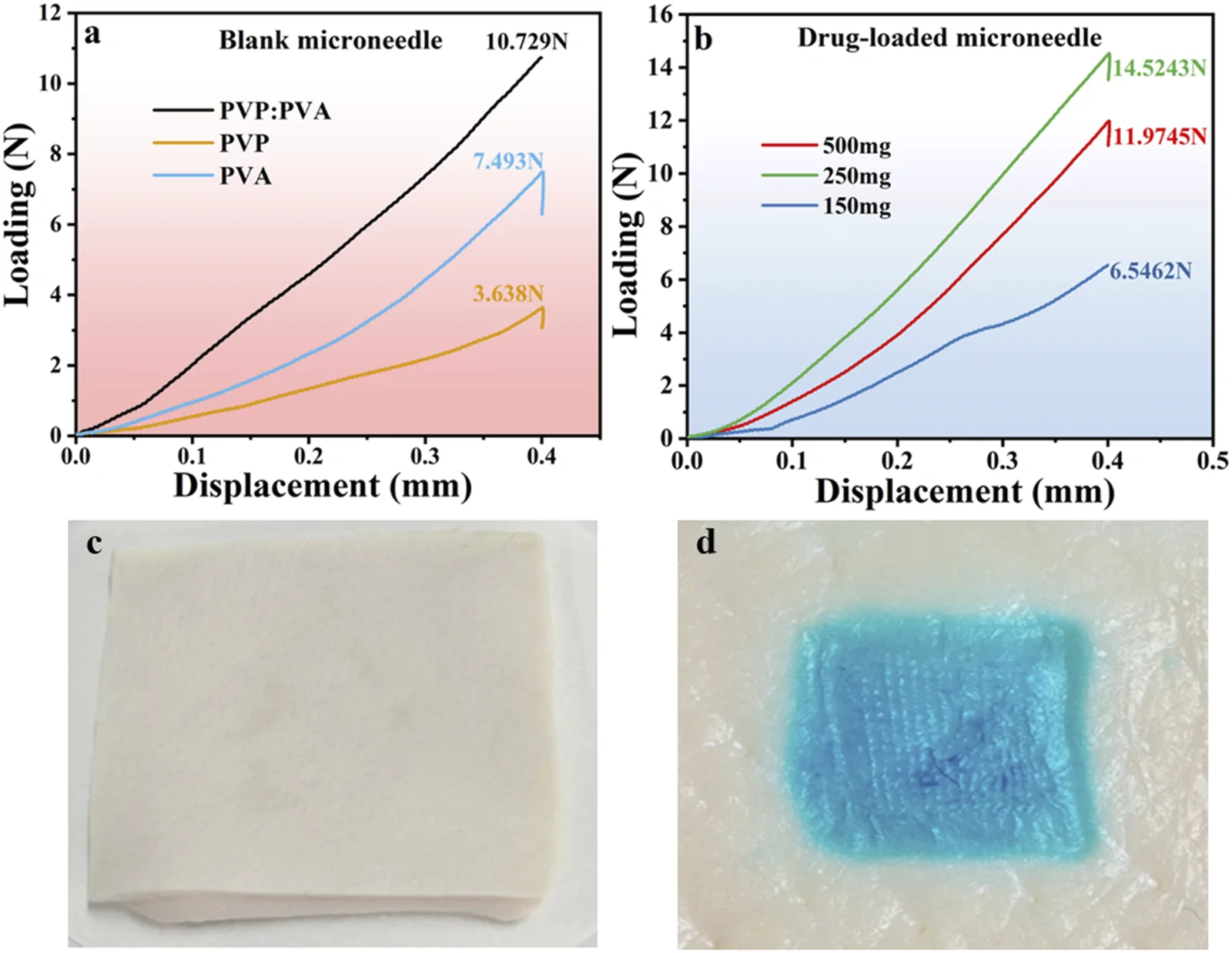
Mechanical and insertion performance of microneedles. (a) Force–displacement curves of blank microneedles prepared from different polymers. (b) Force–displacement curves of drug-loaded microneedles with varying MgH_2_–magnolol release unit contents. (c and d) Optical images of skin before (c) and after (d) microneedle insertion followed by trypan blue staining.

### 3.5 Drug loading and releasing performance of MgH_2_–magnolol-mediated dissolving microneedles

To evaluate the drug loading capacity of the magnesium hydride magnolol polymer microneedles, the absorbance of magnolol standard solutions was measured at 263 nm using ultraviolet-visible spectrophotometry, and a concentration–absorbance calibration curve was established. The linear regression equation was determined to be *y* = 42.32416*x* − 0.00881, with a correlation coefficient of *R*^2^ = 0.99996, indicating excellent linearity within the tested concentration range (Fig. 6a). In this study, a 10 mL stock solution containing 2000 mg of polymer (PVP : PVA = 1 : 1) and varying masses of magnolol was prepared and cast into a microneedle mold to fabricate a total of 20 patches, yielding a polymer matrix mass of 100 mg per patch. The MgH_2_–magnolol complex was prepared at a mass ratio of 1 : 1, such that each component contributes equally to the total complex loading. The actual drug loading of each patch was determined by UV spectrophotometry, with three patches analyzed from each of the three drug-loaded batches. Based on this calibration curve, the actual drug content of microneedles at each theoretical loading level (150, 250, and 500 mg) was quantified. One microneedle was randomly sampled from each of the three independent batches per loading level, yielding a total of nine microneedles for analysis. The actual drug loadings for the three groups were 6.89 ± 0.02 mg per patch for the 150 mg group, 11.48 ± 0.01 mg per patch for the 250 mg group, and 22.96 ± 0.02 mg per patch for the 500 mg group (Fig. 6b). Loading efficiencies were 91.87%, 91.84%, and 91.66%, with slight fluctuations mainly attributed to manual deviations during removal of the drug-loading solution. Despite minor batch-to-batch variation, overall loading remained stable and reproducible. Spectrophotometric quantification, widely used for dissolving microneedles, typically yields calibration curves with correlation coefficients of 0.998 or higher, indicating excellent linearity. The present efficiencies are comparable to reported values of 94.5 ± 1.93% for other dissolving microneedle formulations.

**Fig. 6:**
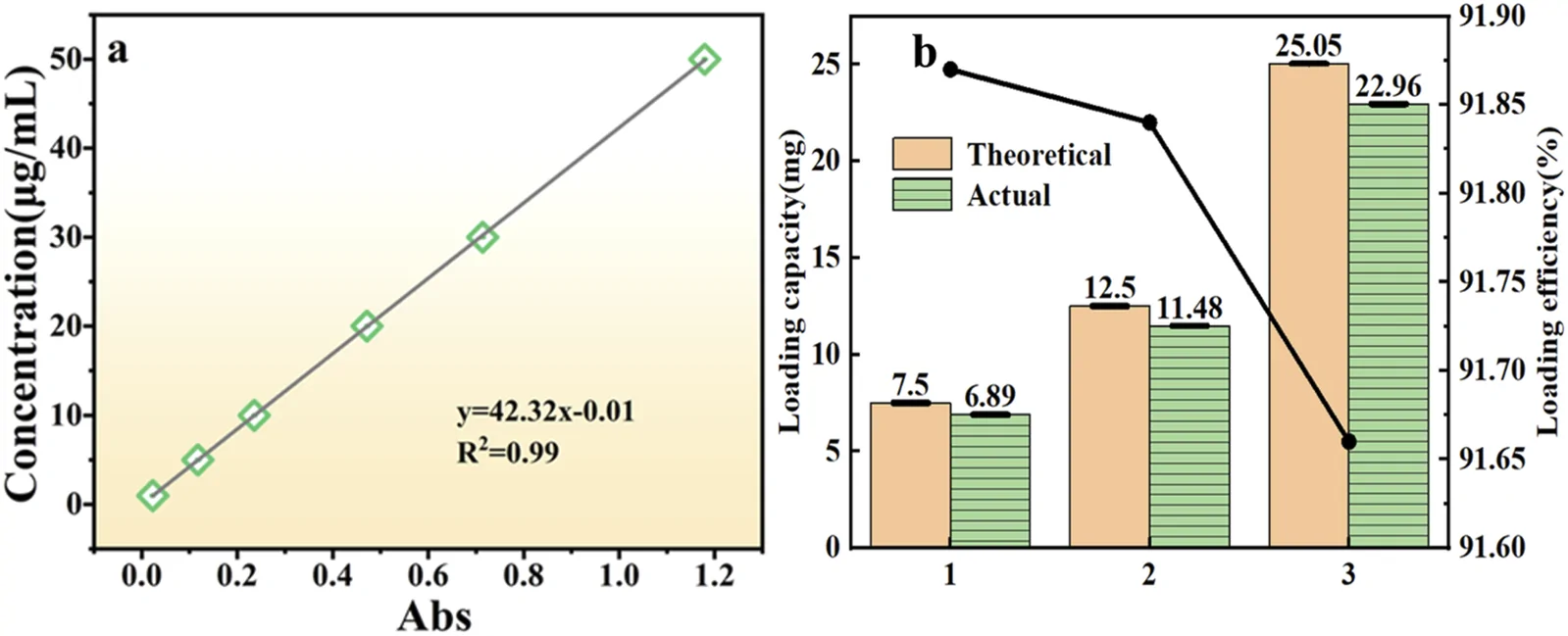
Quantitative characterization of magnolol and MgH_2_–magnolol: (a) magnolol absorbance standard curve, (b) bar graph of loading amount of MgH_2_–magnolol.

To investigate the drug release kinetics of the magnesium hydride magnolol polymer microneedles, a microneedle sample with a drug loading of 150 mg was employed and the cumulative drug release percentage was determined at 10 min intervals to construct the release profile illustrated in Fig. 7. The release rate remained essentially constant during the first 50 min and then declined substantially, with cumulative release reaching 80.7% at 90 min. The rapid initial phase from 0 to 50 min is attributed to direct contact of surface and superficial drug molecules with the release medium, causing swift dissolution and a steep rise in cumulative release. From 50 to 90 min, the slope gradually decreased and release decelerated markedly, because internally entrapped drug molecules must traverse longer matrix diffusion pathways, where increased mass transfer resistance impedes release.

**Fig. 7:**
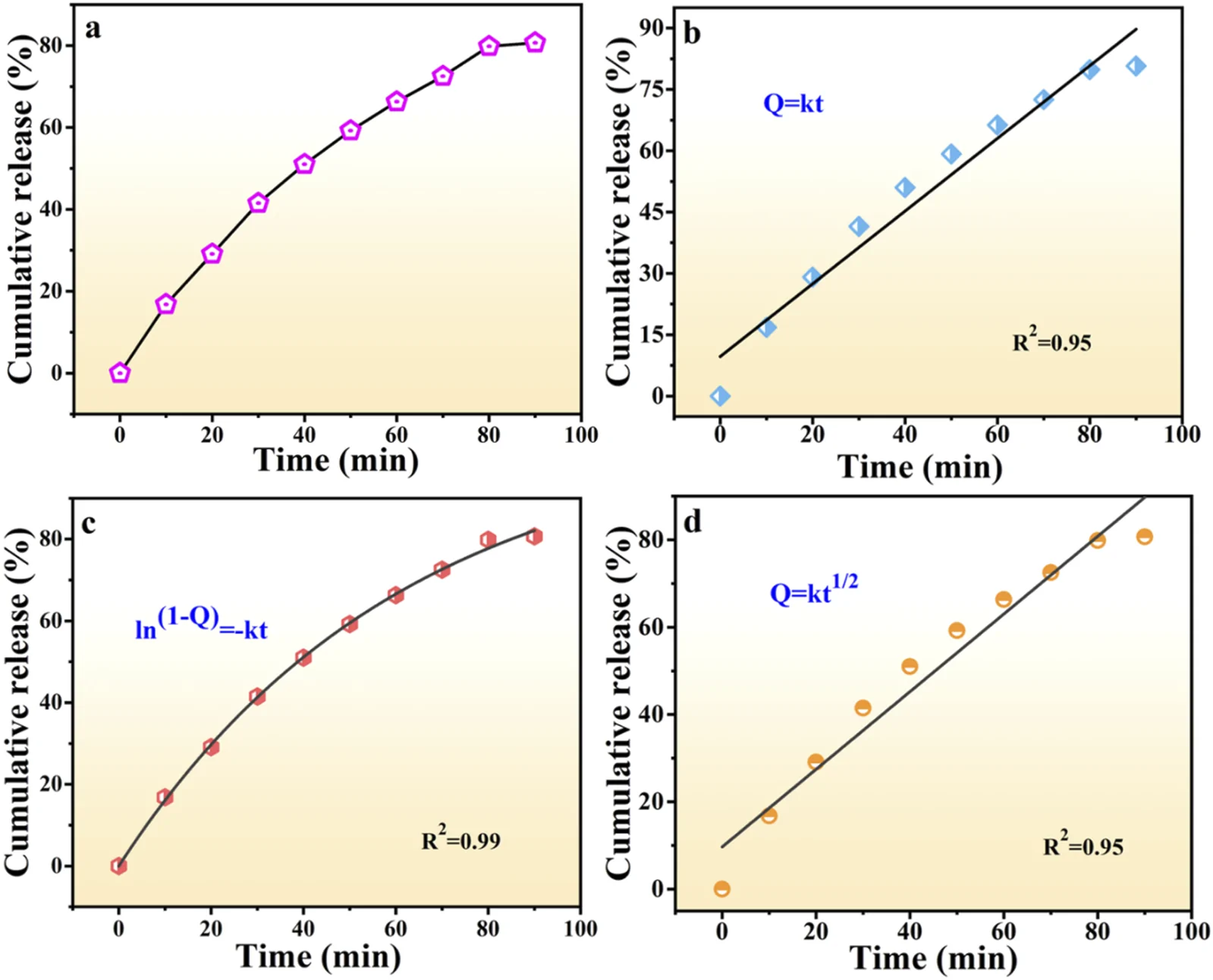
(a) MgH_2_–magnolol polymer microneedle drug release curve, (b) zero-order drug release kinetic equation fitting, (c) first-order drug release kinetic equation, (d) Higuchi drug release kinetic equation fitting.

To elucidate the drug release kinetic mechanism of the magnesium hydride magnolol polymer microneedles, the cumulative release data were fitted to zero-order kinetics, first-order kinetics, and the Higuchi diffusion model, with the original release profile and the corresponding fitting results presented in Fig. 7a through Fig. 7d. The correlation coefficient *R*^2^ shown in Table 1 obtained from zero-order kinetic fitting was 0.95402, indicating that the release process conformed to zero-order kinetics to a certain extent, meaning the drug release rate remained constant over a specific period, presumably because the microneedle architecture furnished relatively stable diffusion channels during the early release phase that enabled uniform drug efflux. The correlation coefficient for first-order kinetic fitting reached as high as 0.99899, exhibiting a far more pronounced correlation and revealing that the release process closely adhered to the first-order kinetic model. This implies that drug release was predominantly governed by a diffusion process driven by the internal drug concentration gradient within the microneedle matrix, such that the concentration gradient diminished progressively with ongoing release and the release efficiency accordingly declined. Similar observations have been reported in dissolving microneedle systems where drug diffusion followed the first-order kinetic model with high correlation coefficients.^43–45^ In contrast, the correlation coefficient for fitting to the Higuchi equation was merely 0.95402, reflecting a goodness-of-fit substantially inferior to that of the first-order kinetic model. In summary, the drug release kinetics of the MgH_2_–Mag polymer microneedles conformed more closely to the first-order kinetic equation, signifying that the drug release rate was primarily modulated by a diffusion mechanism dominated by the concentration gradient within the matrix.

**Table 1:** Fitting of kinetic equations to MgH_2_–magnolol polymeric microneedles

Model	Equation	*R* ^2^	Fitted equation
Zero-order	*Q* = *kt*	0.95402	*Q* = 0.88903*t* + 9.68364
First-order	ln^(1−*Q*)^ = −*kt*	0.99879	*Q* = 106.59924(1 − e^0.01633^*t*)
Higuichi	*Q* = *kt*^1/2^	0.95402	*Q* = 1.77806*t*^1/2^ + 9.68364

Drug release from the MgH_2_–Mag microneedle system was evaluated at four temperatures (Fig. 8a). Cumulative release increased with time at all temperatures, with steeper initial slopes indicating faster release, and showed a clear temperature dependence at identical time points, reaching 96% at 40 °C, 95.8% at 36 °C, 90.3% at 32 °C, and only 80.7% at 25 °C, confirming the distinct temperature sensitivity of magnolol. This acceleration arises from thermal enhancement of drug diffusion and matrix dissolution or swelling, as elevated temperature increases molecular thermal motion and the diffusion coefficient while lowering the activation energy for polymer chain segmental motion, creating a looser matrix and more favorable mass transfer. Thus, drug release can be precisely modulated by adjusting ambient temperature to meet diverse therapeutic requirements. Light conditions markedly affected drug release from the drug-loaded polymeric microneedles (Fig. 8b). Release increased with time under all conditions, with steep initial slopes. UV irradiation produced the highest release (96%) and remained uppermost for most of the interval, whereas dark and ambient light yielded only 78.3% and 80.7%, respectively. Light, especially UV, supplies additional energy that activates drug molecules, accelerates diffusion and matrix egress, and enhances polymer chain segmental mobility, loosening the matrix architecture and facilitating release. Conversely, under dark conditions the absence of photoenergy input results in comparatively sluggish drug molecule diffusion and matrix swelling or degradation, thereby sustaining the release rate at a lower level. Temperature and light constitute key environmental factors that govern the drug release behavior of the drug-loaded microneedles.^46–49^ From the perspective of microneedle architecture and material composition, the characteristics of the internal pore network, the drug–matrix interaction mechanisms, and the stability of the matrix response to photothermal stimuli collectively determine the release properties.^50^ In practical use the release rate can be flexibly regulated according to specific therapeutic demands and environmental conditions to achieve precise drug delivery, while strict control of temperature and light parameters is also imperative during microneedle fabrication and storage to ensure stable and efficacious performance.

**Fig. 8:**
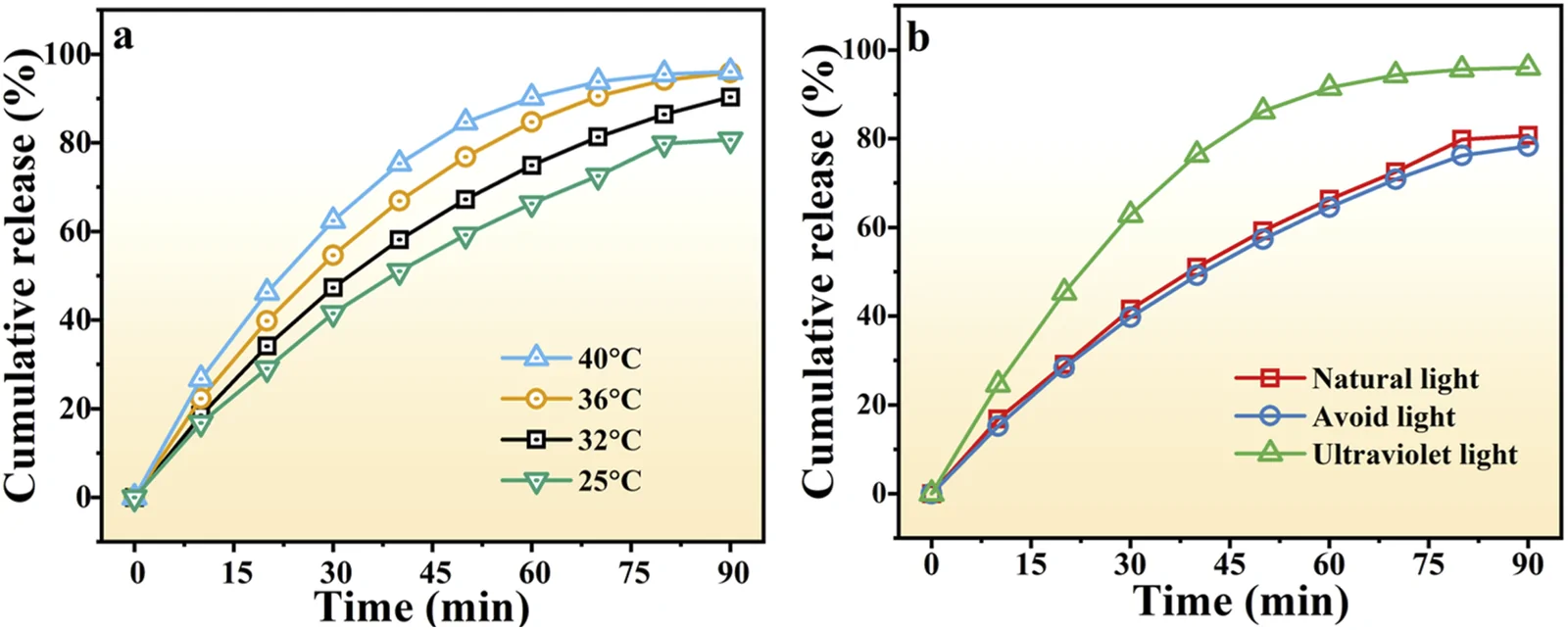
Stimuli-responsive release behavior of MgH_2_–magnolol polymer microneedle system (a) temperature, (b) light.

### 3.6 Drug loading and release mechanism of MgH_2_–magnolol-mediated dissolving microneedles

The drug loading and release mechanism is governed by composite particle formation, diffusion-controlled kinetics, and synergistic therapeutic actions. Mechanical ball milling preserves the crystalline integrity of both magnesium hydride and magnolol, yielding a compact biphasic architecture as confirmed by XRD and FTIR analyses. Upon incorporation into dissolving polyvinyl alcohol polyvinylpyrrolidone microneedles, drug loading enhances mechanical strength from 10.729 N to 14.524 N at 250 mg through two synergistic mechanisms: drug molecules form chemical bonds or physical adsorption interactions with the polymer matrix, creating a reinforcing phase, while simultaneously filling micropores or defects within the material.

The mechanism of the MgH_2_–Mag dissolving microneedle system, as illustrated in Fig. 9, can be re-described as a cascade of composite formation, interfacial reinforcement, diffusion-controlled release, environmental responsiveness and synergistic therapy. Ball milling preserves the crystalline integrity of both MgH_2_ and magnolol, yielding a compact biphasic architecture that, after loading into dissolving polyvinyl alcohol–polyvinylpyrrolidone (PVA–PVP) microneedles, raises mechanical strength from 10.729 to 14.524 N at 250 mg through interfacial bonding or physical adsorption with the polymer matrix and simultaneous micropore filling. Drug release is predominantly diffusion-controlled and follows first-order kinetics most closely (*R*^2^ = 0.99899), indicating concentration-gradient-driven transport.^29,30^ In the initial 0 to 50 min, surface-associated drug dissolves rapidly, whereas from 50 to 90 min internally entrapped molecules must traverse increasingly tortuous matrix pathways, where mass transfer resistance slows release; zero-order and Higuchi fits (*R*^2^ = 0.95402) confirm that polymer dissolution and matrix erosion contribute but are not rate-limiting.^31^ The system also displays environmental responsiveness, since elevated temperature enhances molecular thermal motion and ultraviolet irradiation supplies additional energy that activates drug molecules and increases matrix segmental mobility, achieving up to 96% cumulative release at 40 °C or under ultraviolet light *versus* 80.7% under ambient conditions.^51,52^ In parallel, MgH_2_ reacts with bodily fluids to generate H_2_ and Mg^2+^ in a sustained manner, adding multi-responsiveness and locally altering matrix hydration and dissolution. Therapeutically, MgH_2_ particles scavenge excessive reactive oxygen species and relieve inflammation by inhibiting the NF-κB pathway while downregulating IL-1β, IL-6, and TNF-α, and Mg^2+^ promotes M2-type macrophage polarization and angiogenesis for tissue repair.^53,54^ Magnolol suppresses MAPK phosphorylation, activates Nrf2 nuclear translocation with upregulation of HO-1, NQO1, and Keap1, and inhibits NLRP3 inflammasome activation to reduce oxidative stress and inflammation.^55–57^. The integration of these complementary agents within a mechanically robust and environmentally tunable microneedle platform supports this system as a promising minimally invasive strategy for androgenetic alopecia treatment.

**Fig. 9:**
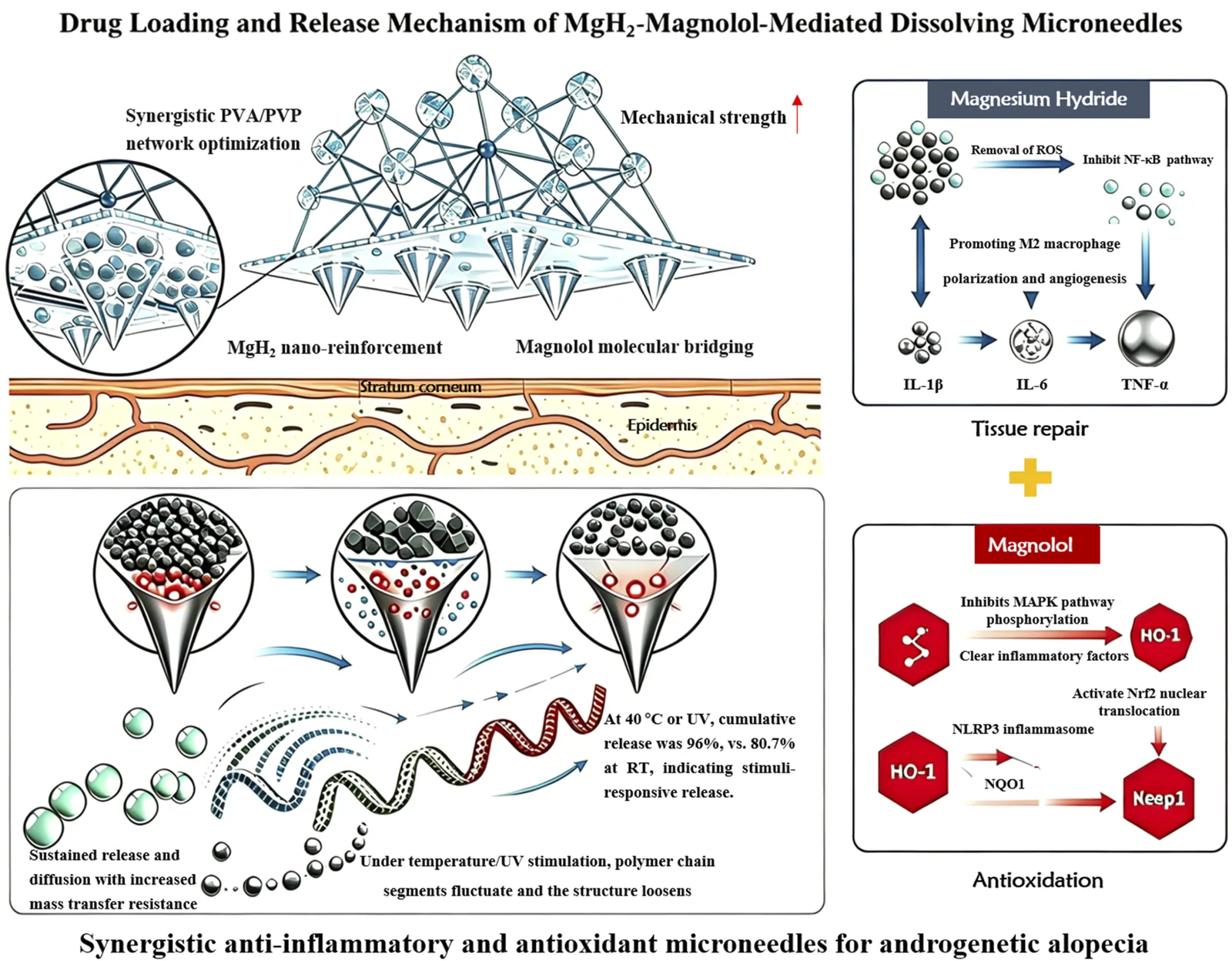
Drug loading and release mechanism of MgH_2_–magnolol-mediated dissolving microneedles.

## 4. Conclusions

(1) Ball milling preserved the crystalline phases of MgH_2_ and magnolol and produced a compact, uniform composite, whereas ethanol solvent deposition caused magnolol amorphization or high dispersion, as confirmed by XRD and FTIR.

(2) The PVA:PVP (1 : 1) microneedles exhibited a fracture force of 10.729 N, sufficient for skin insertion, which increased to 14.524 N at 250 mg drug loading through matrix reinforcement and defect filling.

(3) Drug release followed first-order kinetics (*R*^2^ = 0.99899), indicating diffusion- and concentration-gradient-controlled release, and was accelerated by elevated temperature or UV irradiation, reaching 96% cumulative release at 40 °C or under UV light *versus* 80.7% under ambient conditions.

(4) The MgH_2_–Mag dissolving microneedle system integrates phase integrity, mechanical robustness, and tunable release, supporting its potential as a minimally invasive strategy for androgenetic alopecia, while further safety and *in vivo* efficacy studies are warranted.

## Conflicts of interest

The authors report no conflicts of interest in this work.

## Data Availability

All data generated or analysed during this study are included in this published article.
